# Assisting during microsurgery: important first steps

**Published:** 2023-12-01

**Authors:** Jacqueline Newton

**Affiliations:** 1 Staff Nurse: Flying Eye Hospital, Orbis International, Cape Town, South Africa.

**Scrub nurses or technicians can prepare for their role by learning about instruments and instrument trays, learning the steps of procedures, and reviewing surgical procedures with the surgeons they will assist.**

**Figure:**
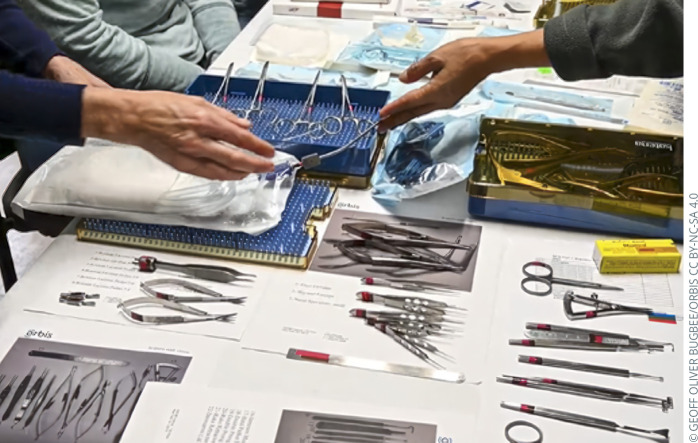
Learning about different surgical instruments and what they are used for is an important first step. **CHILE**

## 1. Learn about surgical instruments and trays

The first step in learning to assist during microsurgery is to learn the names of the instruments and to become familiar with the different types of instrument trays.

A good place to start is in the sterilisation area, where instruments are reprocessed and packed for sterilisation in preparation for surgery. While you are in the sterilisation area:

- Use an ophthalmic instrument catalogue to identify the different instruments
- Use a magnifying glass to look at the fine tips of hooks or lens manipulators.

**Note**

Ophthalmic microsurgical instruments are *very* delicate and have fine tips that need to be handled with great care.

Each type of surgery or ophthalmic subspecialty (e.g. cataract, squint, cornea, oculoplastic) has a different instrument tray, containing the instruments the surgeon will need. Instruments on each of these trays are categorised into:

- Forceps and clamps (forceps can be toothed, smooth, serrated, or micro-notched; straight, curved, or angled)
- Scissors (sharp or blunt tips; straight, curved, or angled)
- Needle holders (locking or non-locking; straight or curved)
- Hooks, lens manipulators, retractors, and loops
- Cannulated instruments, e.g., Simcoe and anterior chamber cannulas
- Blade handles
- Speculums, calipers, or rulers.

Ask a senior colleague to show you what each instrument tray should look like. Take a photograph or draw the tray, then label each instrument. Ask your colleague to check everything is correct and in place.

**Teaching tip**

**Figure 1:**
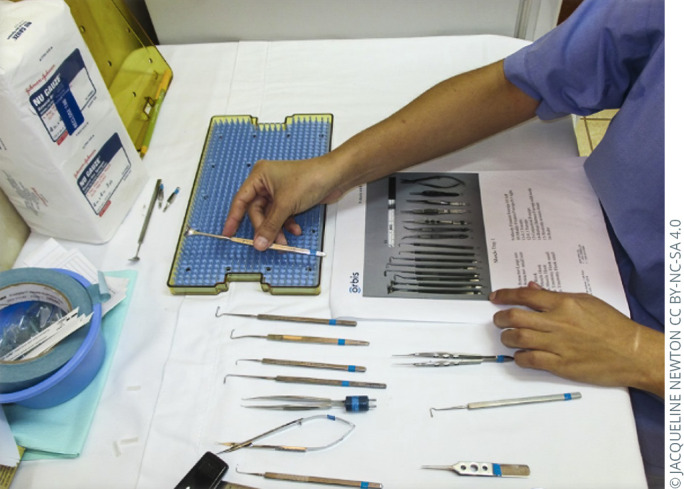
A nurse practices arranging microsurgical instruments on a tray before sterilisation. **PERU**

Create a photograph or drawing of each type of instrument tray and label each instrument with its name. Allow the trainee nurse or technician to arrange the trays accordingly, in preparation for sterilisation (Figure 1).

## 2. Learn the steps of procedures

An excellent and safe way of preparing for a procedure is to learn the surgical steps and practise for them through simulation.

**Figure 2:**
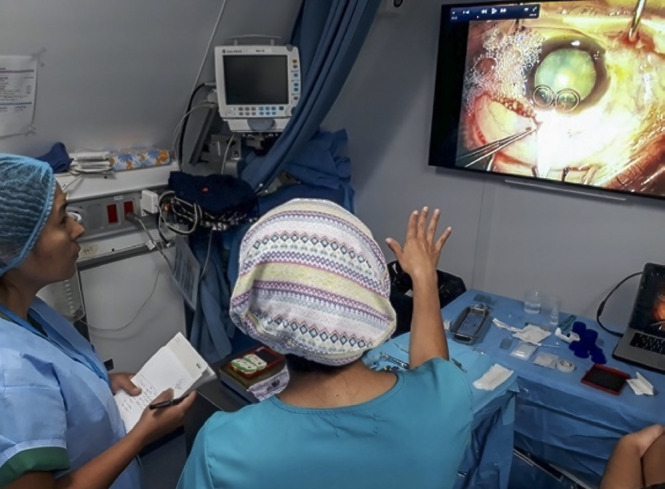
Nurses watch an operation being performed and note down the surgical steps, in order. **PERU**

**Figure 3:**
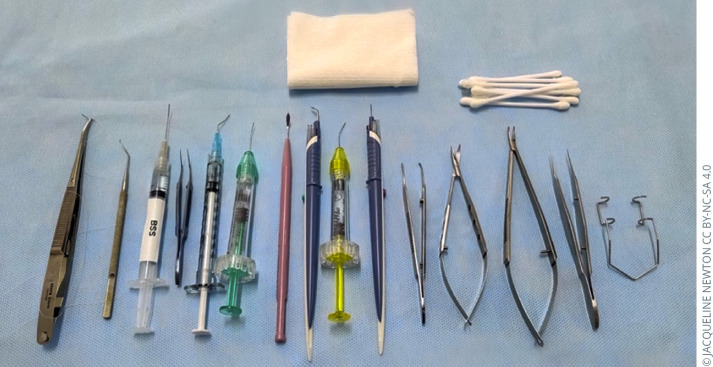
Setting up surgical instruments in simulation.

- Watch a video recording of the operation to be performed, or observe a live operation (Figure 2). Write down each surgical step and the instruments and supplies used. You can make a checklist, in table form (see Table 1).
- Next, arrange the surgical instruments on a simulated/mock ‘sterile field’ in the same order as the surgical steps (Figure 3).
- If you have a smartphone, take a photo of the setup to remind yourself of the sequence of steps.

**Table 1:** A checklist summarising the steps of small-incision cataract surgery and the supplies and instruments needed.

Description of the step	Consumables/supplies	Surgical instrument
Placing speculum		Type:
Place bridal/stay suture	Suture:	Superior rectus forceps Needle holder Clamp
Dissect conjunctiva		Blunt curved Westcott scissors Non-toothed forceps
Cautery to blood vessels	Eraser tip:	Cautery cable/cautery forceps
First scleral incision	Blade:	Fine-toothed forceps
Scleral tunnel	Blade:	Fine-toothed forceps
Paracentesis	Blade:	Fine-toothed forceps
Stain the anterior capsule	Vision blue/air bubble	
Flush with BSS	2cc syringe & 27G cannula	
Viscoelastic injection	Type:	
Main sclerocorneal incision	Blade:	Fine-toothed forceps
Capsulotomy	Cystotome	Capsulorhexis forceps
Placement of AC maintainer	AC maintainer	
Hydrodissection	2cc syringe 27G hydrodissection cannula	
Enlarge main incision	Blade:	Fine-toothed forceps
Remove nucleus		Vectus loop
Cortex removal	Simcoe and IV tubing 5 or 10cc syringe	
Viscoelastic	Type:	
Insertion of IOL	IOL:	McPherson forceps Holding & folding forceps
Dial the IOL in place		Sinsky hook
Remove viscoelastic	Simcoe and IV tubing 5 or 10cc syringe	
Hydrate wounds (BSS)	2cc syringe & 27G cannula	
Check wounds for leakage	Microsponge	
Close conjunctiva	Suture:	Cautery cable/cautery forceps
Subconjunctival injection	1cc syringe & 30G needle	
Remove bridal/stay suture		Scissors
Eyedrops/ointment		
Eye pad/eye shield		

## 3. Review procedures with the surgeon

A scrub nurse or technician may work with a different surgeon every day. It is helpful to set aside time to review the basic steps of the next day's surgical procedures with the surgeon, as this will help you to anticipate their needs more effectively.

I found it helpful to create a checklist, in table form, for the most common operations I'm likely to be involved in (Table 1), listing the surgical step, the type of consumable the surgeon may use, and the type of surgical instrument the surgeon may use.

Reviewing these steps with the surgeon may feel daunting at first, but you will become more confident the more you do it. When surgeons see that you are interested in being prepared, they will usually be happy to share this information, as it supports collaboration between surgeon and nurse, which can lead to better outcomes for patients.

**Expert tip**

I found it helpful to create basic checklists, in table form, for the most common operations I'm likely to be involved in. Table 1 shows an example of the table I created for small-incision cataract surgery (SICS). There is space to write note about the surgeon's preferences, such as the type of blade or suture they plan to use.

